# Blood and milk metabolites of Holstein dairy cattle for the development of objective indicators of a subacute ruminal acidosis

**DOI:** 10.5713/ab.22.0486

**Published:** 2023-02-26

**Authors:** Hyun Sang Kim, Jun Sik Eom, Shin Ja Lee, Youyoung Choi, Seong Uk Jo, Sang Suk Lee, Eun Tae Kim, Sung Sill Lee

**Affiliations:** 1 Institute of Agriculture and Life Science, Gyeongsang National University, Jinju 52828, Korea; 2 University-Centered Labs, Gyeongsang National University, Jinju 52828, Korea; 3 Division of Applied Life Science (BK21), Gyeongsang National University, Jinju 52828, Korea; 4 Ruminant Nutrition and Anaerobe Laboratory, College of Bio-industry Science, Sunchon National University, Suncheon 57922, Korea; 5 National Institute of Animal Science, Rural Development Administration, Cheonan 31000, Korea

**Keywords:** Concentrate, Dairy Cow, Forage, Metabolite, Proton Nuclear Magnetic Resonance, Ruminal Acidosis

## Abstract

**Objective:**

The purpose of this study was to perform a comparative analysis of metabolite levels in serum and milk obtained from cows fed on different concentrate to forage feed ratios.

**Methods:**

Eight lactating Holstein cows were divided into two groups: a high forage ratio diet (HF; 80% Italian ryegrass and 20% concentrate of daily intake of dry matter) group and a high concentrate diet (HC; 20% Italian ryegrass and 80% concentrate) group. Blood was collected from the jugular vein, and milk was sampled using a milking machine. Metabolite levels in serum and milk were estimated using proton nuclear magnetic resonance and subjected to qualitative and quantitative analyses performed using Chenomx 8.4. For statistical analysis, Student’s t-test and multivariate analysis were performed using Metaboanalyst 4.0.

**Results:**

In the principal component analysis, a clear distinction between the two groups regarding milk metabolites while serum metabolites were shown in similar. In serum, 95 metabolites were identified, and 13 metabolites (include leucine, lactulose, glucose, betaine, etc.) showed significant differences between the two groups. In milk, 122 metabolites were identified, and 20 metabolites (include urea, carnitine, acetate, butyrate, arabinitol, etc.) showed significant differences.

**Conclusion:**

Our results show that different concentrate to forage feed ratios impact the metabolite levels in the serum and milk of lactating Holstein cows. A higher number of metabolites in milk, including those associated with milk fat synthesis and the presence of *Escherichia coli* in the rumen, differed between the two groups compared to that in the serum. The results of this study provide a useful insight into the metabolites associated with different concentrate to forge feed ratios in cows and may aid in the search for potential biomarkers for subacute ruminal acidosis.

## INTRODUCTION

Dairy cows are fed high-quality forage to increase milk yield. However, insufficient energy metabolism in the body can negatively impact body weight (BW) and milk yield [1]. Concentrates containing high starch and crude protein (CP) are important in ruminant nutrition, as they are a practical source of energy and have been shown to influence rumen function, such as potential effects on ruminal ammonia and nitrogen utilization and on amino acid supply for microbial protein synthesis, consequently affecting milk protein synthesis and milk yield in high-producing dairy cows [2]. The ratio of concentrate to forage in feed should be adjusted considering the physical and chemical properties of the concentrate, the digestibility of the feed, and the stage of lactation, and this is being explored extensively [3].

High-grain diets increase the concentration of organic acids, such as volatile fatty acids, in the rumen due to rapid fermentation and decreased ruminal pH, causing ruminal acidosis [4]. This can compromise rumen microbial populations and the rumen mucosa; cause laminitis, diarrhea, and liver abscesses; and increase enterohemorrhagic *Escherichia coli* (*E. coil*) in dairy cows [5]. Therefore, it is necessary to study the metabolic changes related to ruminal acidosis that are triggered by diet in various body fluids such as blood (plasma and serum), urine, milk, and rumen fluid.

Proton nuclear magnetic resonance (^1^H-NMR) is the preferred technology for metabolomic studies due to its exceptional capacity to handle complex metabolite mixtures [6]. In addition to this, ^1^H-NMR has several unique advantages: it requires simple pretreatment, allows for easy quantification, requires no separation, and permits the identification of compounds. However, ^1^H-NMR is a relatively insensitive technique and requires a large sample volume. Recent improvements such as larger field magnets, cryogenically cooled probes (that increase signal by a factor of three), and small-volume microprobes (60 μL) have been introduced to increase the analytical power of this technique [7].

In the previous study, rumen microbial communities and abnormal metabolites for subacute ruminal acidosis in dairy cows are being conducted, and biochemical analysis of blood and changes in fatty acid profile in milk are being studied. Therefore, the purpose of this study is to provide a theoretical basis for the change of metabolite in serum and milk for the prevention of subacute ruminal acidosis in dairy cows.

## MATERIALS AND METHODS

This study was carried out in strict accordance with the recommendations in the Guide for the Care and Use of Laboratory Animals of the National Institute of Animal Science in Korea. The protocol was approved by the Committee on the Ethics of Animal Experiments of the National Institute of Animal Science (NIAS-2017-249).

### Animals, experimental design, and dietary treatments

Eight lactating Holstein cows (BW, 598±77 kg; parity, 1 to 3; days since calving, 168±20 days; milk yield, 24.49 kg/d) were divided in two treatment groups: a high-forage diet (HF, 10 kg; Italian ryegrass 80%:concentrate 20%) group and a high-concentrate diet (HC, 14.2 kg; Italian ryegrass 20%:concentrate 80%) group. Concentrate to forage ratio was calculated according to the BW of dry matter (DM) that was provided to the cows twice a day. The animals were also given *ad libitum* access to mineral blocks and water twice daily, approximating 5% of the total feed ingested. Details of ingredients and chemical composition of the diets are given in Supplementary Table S1. The contents of DM (method No. 934.01), CP (method No. 976.05), calcium (method No. 927.02), and phosphorus (method No. 3964.06) in Italian ryegrass and concentrate were assayed as described by Association of Official Analytical Communities methods [8]. The rumen pH of HC group and HF group was monitored for 6 consecutive hours after feeding in the morning every day. The HF group confirmed that the appropriate pH range was maintained, and HC group make sure the pH was below 5.5 for more than 3 h. The experiment lasted 14 days, with first 7 days as the diet adaptation period [9].

### Blood and milk sampling and proton nuclear magnetic resonance preparation

Blood and milk sampling were obtained from 4 h after feeding in the morning. Blood samples were collected from the jugular vein suing a 22G needle connected to a 20 mL syringe. All blood samples were collected into 8.5 mL vacutainer tubes and centrifuged for 5 min at 15,140×g. The serum, which is the supernatant, was separated and stored at −80°C until analysis. A saline buffer with NaCl 0.9% weight/volume in 100% deuterium oxide (D_2_O) was prepared. The stored serum sample was centrifuged at 14,000×g for 10 min at 4°C. The supernatant (200 μL) was added to 400 μL of saline buffer in the 5 mm NMR tube for measurement. An ERETIC peak reference sample was also prepared by adding 388 μL of saline solution and 12 μL of 100 mM valine solution to 200 μL of the supernatant to yield a final valine concentration of 2 mM, which was then transferred to the 5 mm NMR tube.

Milk sample was collected 50 mL conical tube containing preservative (Broad Spectrum Microtabs II) from individual animals using a milking machine. The thawed raw milk samples were centrifuged at 4,000×g for 15 min at 4°C to obtain skim milk. The clear liquid sample were conserved −80°C until further analysis. The collected milk sample was centrifuged at 4,000×g for 15 min to remove the lipid layer in the supernatant. Thereafter, the mixture of milk (250 μL) and D_2_O (300 μL) was transferred to a 5 mm NMR tube for analysis [10].

### Proton nuclear magnetic resonance analysis

The samples were analyzed using an Ascend 800 MHz, AVANCE III HD Bruker spectrometer (Bruker BioSpin AG, Fällanden, Switzerland). Proton nuclear magnetic resonance spectra were acquired at 25°C using the first transient the Carr-Purcell-Meiboom-Gill pulse sequence with the following parameters: temperature = 25°C, repetition number = 128, acquisition time = 2.0 s. The free induction decay was acquired with a spectral width of 20 ppm and collected to 64 k data points.

### The proton nuclear magnetic resonance data processing and statistical analyses

The spectra of milk and serum were assigned using Chenomx NMR Suite 8.4 software (Chenomx Inc, Edmonton, AB, Canada). The phase and baseline of the spectra were corrected using the Chenomx processor, and the qualitative and quantification of metabolites were performed using the Chenomx profiler. The region of the spectrum that included water (δ 4.6 to 5.0) was removed from the analysis for all spectra files. The spectral width was 10 ppm and was referenced to the 3-(trimethylsilyl)propionic-2,2,3,3-d_4_ acid (TSP) signal at 0 ppm. The metabolite databases used for classification were the Bovine Metabolome Database (www.bovinedb.ca), Livestock Metabolome Database (www.lmdb.ca), and the Human Metabolome Database (www.hmdb.ca).

The metabolites of milk and serum were compared between the groups using unpaired Student t-test by Metaboanalyst 4.0 (www.metaboanalyst.ca) based on *R* software and the changes with p-value <0.05 were considered as statistically significant and 0.05<p<0.1 was considered to indicate a tendency toward a significant difference. Multivariate analysis including principal component analysis (PCA), partial least squares-discrimination analysis (PLS-DA), orthogonal partial least squares-discriminant analysis (OPLS-DA), and variable importance in projection (VIP) scores were performed using Metaboanalyst 4.0. The corresponding VIP scores were figured in the supervised PLS-DA model to evaluate the recognizing power of the identified metabolite. Differences in metabolite abundance were assessed by heat map visualization analysis employing the logarithms of metabolite quantification for each sample replicate.

## RESULTS

### Metabolites identified in serum and milk

**Figure 1:**
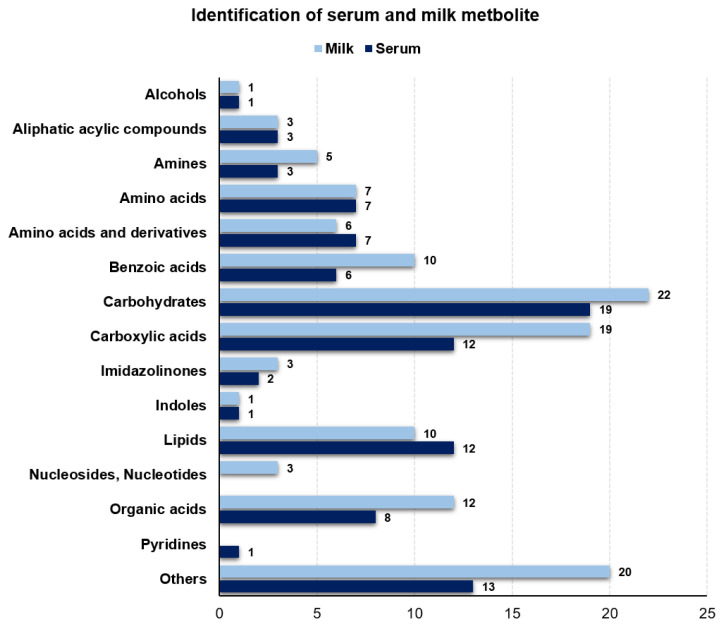
The classification of the total measured of metabolite in serum and milk. Identified metabolite are categorized according to chemical class and number of metabolite detected by proton nuclear magnetic resonance.

We performed metabolite profiling, using ^1^H-NMR analysis, of serum and milk from diary cows on diets with different concentrate to forage feed ratios. Classifications of metabolites, including alcohols, aliphatic acyclic compounds, amines, amino acids, amino acids and derivatives, benzoic acids, carbohydrates, carboxylic acids, imidazolinones, indoles, lipids, nucleosides and nucleotides, organic acids, pyridines, and others in serum and milk were quantified. We identified a total of 122 and 95 metabolites in milk and serum, respectively (Figure 1).

### Multivariate analyses

**Figure 2:**
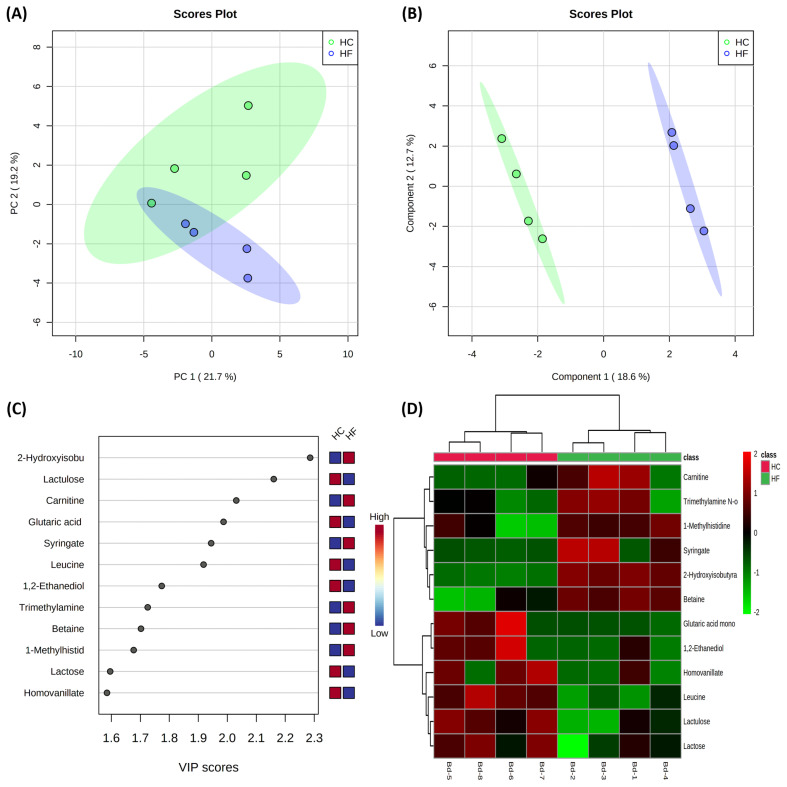
Multivariate analysis on serum of high concentrate (HC) and high forage (HF) groups. Principal component analysis (A); Partial least squares-discriminant analysis (PLS-DA) (B); Variable importance in projection (VIP) analysis of top 12 metabolites (score value >1.5) (C). Heat map for the metabolite profiles is displayed based on top 12 metabolites identified by PLS-DA VIP score (D). Ellipse represents 95% confidence interval.

Results of the multivariate statistical analysis of serum metabolites are shown in Figure 2. The PCA revealed that PC axes 1 and 2 accounted for 21.7% and 19.2% of the total variation, respectively (Figure 2A). The PLS-DA score plots revealed that component axes 1 and 2 accounted for 18.6% and 12.7% of the total variation, respectively (Figure 2B). Principal component analysis illustrated a moderate similarity among animals with different concentrate to forage feed ratios. The VIP scores and the corresponding heat map of the top 12 variables of importance are presented in Figures 2C and D, respectively.

**Figure 3:**
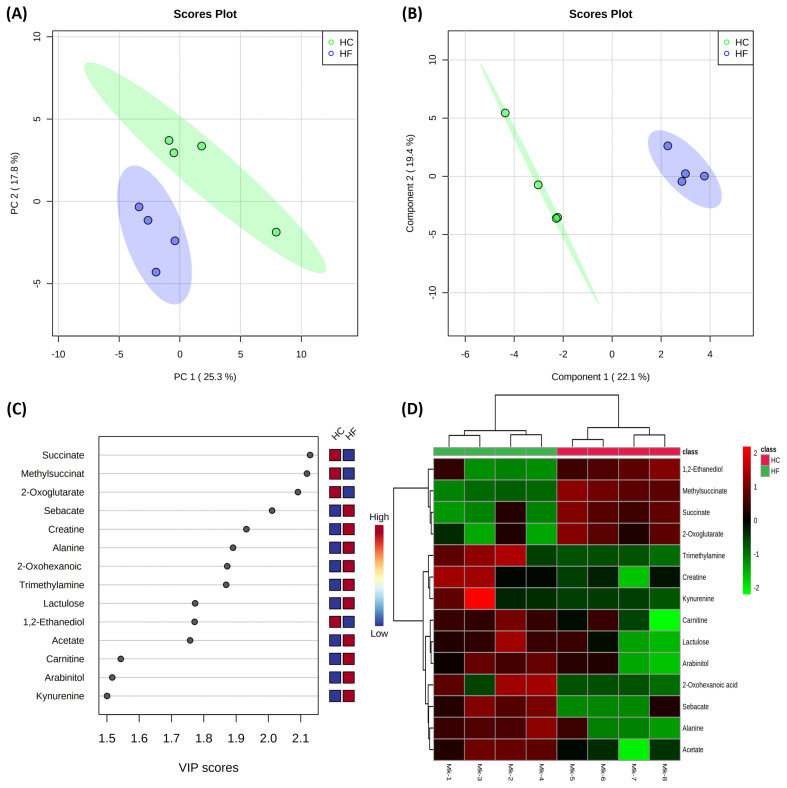
Multivariate analysis on milk of high concentrate (HC) and high forage (HF) groups. Principal component analysis (A); Partial least squares-discriminant analysis (PLS-DA) (B); Variable importance in projection (VIP) analysis of top 14 metabolites (score value >1.5) (C); Heat map for the metabolite profiles is displayed based on top 14 metabolites identified by PLS-DA VIP score (D). Ellipse represents 95% confidence interval.

Results of the multivariate statistical analysis of milk metabolites are shown in Figure 3. The PCA revealed that PC axes 1 and 2 accounted for 25.3% and 17.8% of the total variation, respectively (Figure 3A). The PLS-DA score plots revealed that component axes 1 and 2 accounted for 22.1% and 19.4% of the total variation, respectively (Figure 3B). As expected, the PCA and PLS-DA both showed that the milk metabolites of the HC group were significantly different from those of the HF group. The VIP scores and the corresponding heat map of the top 14 VIP in Figures 3C and D, respectively.

**Figure 4:**
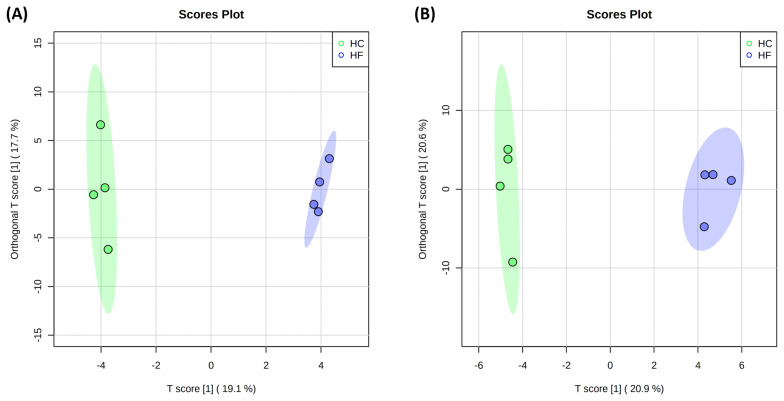
Orthogonal partial least squares-discriminant analysis of serum (A) and milk (B) metabolite. Ellipse represents 95% confidence interval.

Orthogonal PLS-DA was used to visualize the discrimination in the serum and milk samples between the HC and HF groups (Figure 4), and it illustrated two distinct groups among animals fed with differing concentrate to forage feed ratios.

### Concentrate to forage feed ratios impact the metabolic profiles of serum and milk in lactating cows

Serum and milk metabolites that were significantly different between both diets were classified according to compound name, and the average concentration, p-value, and VIP score of each metabolite are presented in Table 1 and 2. We identified 13 metabolites, including glucose, carnitine, and syringate that were shared by all serum samples. In addition, betaine, 2-hydroxyisobutyrate, and syringate were significantly higher, whereas citrate, 1-methylhistidine, 3-methylhistidine, and carnitine tended to be higher in the serum of the HF group than in the HC group (Table 1).

Similarly, we identified 20 metabolites, including arabinitol, alanine, acetate, and creatine, that were shared by all milk samples. Methylsuccinate, 2-oxoglutarate, succinate, 1,2-ethanediol, and urea were significantly higher, whereas glucose tended to be higher in the milk of the HC group than that in the HF group (Table 2).

## DISCUSSION

To compensate for the requirement for energy during early lactation, dairy herds consume more grain, increasing their DM intake compared to that of normal circumstances. Consumption of large amounts of enriched diets affects rumen fermentation, placing cows at a high risk of sub-acute ruminal acidosis [11]. The physical form of the feed ingredients is important in determining how quickly and completely the feed is fermented in the rumen. Finely ground, steam flaked, and extruded feed ferment faster and more completely in the rumen than raw and dry grains, even though the chemical compositions of the feeds may be identical [12]. Delineating the consequences of the feed ratio on the metabolite production in dairy cows may provide critical information for understanding the factors impacting milk yield. Our previous study reported metabolite changes in rumen fluid, urine, and feces induced by subacute ruminal acidosis due to a highly concentrated diet [13].

Previous studies have shown that changes in the concentrations of leucine,1,2-ethanediol, acetate, and butyrate in milk and serum might be an indicator of ruminal acidosis [14–16]. Leucine is a branched-chain amino acid that is especially high in corn-based feed and is known to stimulate muscle protein synthesis [17]. Previously, increasing the proportion of cereal grain in feed led to an increase in serum levels of leucine, which is similar to that seen in the HC group compared to that in the HF group [18]. We found an increase in the concentration of syringate in the serum of HF group compared to that in the HC group. Syringate, in addition to vanillate, is a major intermediate metabolite generated during microbial degradation of lignin [19]. Anaerobic degradation of the O-demethylated derivatives of syringate, namely gallate, has been documented in sulfate-reducing, nitrate-reducing, and fermentative bacteria. The increased concentration of syringate in the serum of the HF group is possibly due to the decomposition of bacteria in the rumen due to the high neutral detergent fiber content of the diet. Urea is the end product of the protein catabolic reaction in the body [20]. The urea concentration in milk was significantly higher in the HC group than in the HF group. The concentration of urea and nitrogen in milk is affected by CP intake and digestibility [21]. Ammonia in the rumen is synthesized from urea in the liver and accumulates in the serum and milk. We also found that 1,2-ethanediol (or ethylene glycol) and arabinitol produced by microbial metabolism in the rumen were significantly higher in the milk of the HC group than the HF group. Arabinitol (or arabitol), a sugar alcohol, is produced by pentose and hexasaccharides decomposing yeast [22]. However, 1,2-ethanediol is metabolized by alcohol dehydrogenase to multiple acidic compounds, mainly glycolic acid, which induces metabolic acidosis [23]. It is also produced from xylose, which comprises a major part of the carbohydrates in roughage and cereals, by an enzyme secreted by *E. coli* [15]. Previously, it has been reported that high grain diet-induced subacute ruminal acidosis is associated with an increase in *E. coli* in the rumen [24]. Therefore, correlation between 1,2-ethanediol and microorganisms was studied; this can be used as the potential biomarker of subacute ruminal acidosis. We also found that the concentration of acetate and butyrate in milk of the HF group was higher than that in the HC group. Acetate and butyrate are short-chain fatty acids that are typically used in the synthesis of milk fat and are produced by fermentation of forage by microorganisms in the rumen [25]. These can also originate from body fat mobilization and fat metabolism in the liver. This metabolite results indicated a correlation between forage intake and milk fat synthesis.

Numerous studies have shown comparable results for change of metabolite (carnitine, glucose, and lactulose) in milk and serum in relation to ruminal acidosis. Betaine, carnitine, glucose, and lactulose are metabolites commonly observed in serum and milk [26–28]. Carnitine is present in all animal species, many microorganisms, and many plants and plays several important functions related to metabolism in the body, including the importation of long-chain fatty acids into the mitochondria for β-oxidation via interaction with coenzyme A, which is a functional group attached to fatty acids [29]. Carnitine shows the highest association with the fatty acid, acetate, followed by propionate and butyrate. Due to the nature of the substrate (mainly structural carbohydrates), the rumen turnover and the ratio of electron donors to acceptors is low. In forage-fed ruminants, bacterial species that convert carbohydrates to acetate multiply at the expense of propionate fermenters [30]. In this study, we validated the correlation between the concentration of carnitine in the serum and milk of the HF group. Betaine exists naturally in plants and is the oxidation product of choline and trimethylated derivatives of glycine [31]. Betaine’s functions include volume-keeping osmolality, is a methyl donor that increases methionine and reduces homocysteine concentration and is fermented as acetate in the rumen. In dietary studies, milk fat, milk yield, and fatty acid synthesis were found to increase with an increase in the level of betaine in diet. Our study revealed differences in glucose clearance rate with regard to diet, as indicated by comparable glucose levels in the serum of the HC group, but a trend for a greater decrease in glucose in the HF group. Several studies have observed a decline in mammary extraction of glucose at very high plasma glucose concentrations [32].

In this study, ^1^H-NMR-based metabolomics revealed that levels of several metabolites differed in the serum and milk according to the concentrate to forage feed ratio. In milk, metabolites that differed significantly according to feed ratio were more diverse than those in serum. We found significantly higher concentrations of betaine, carnitine, acetate, butyrate, and arabinitol in the milk of the HF group than in the HC group. On the contrary, glucose and 1,2-ethanediol were significantly higher in the milk of the HC group than in the HF group. These metabolites are closely related to rumen microbial metabolism, milk quality, and metabolic acidosis. Further studies are needed to analyze the microbiome and the effect of diet on the microbial community in the rumen. Such studies will provide important insights into the mechanism underlying ruminal acidosis, and the discovery of metabolite markers of acidosis will aid in its diagnosis without the requirement for blood or rumen fluid sampling from cows.

## Figures and Tables

**Table 1:** Identification of significantly different metabolites (mean±standard deviation) in serum between dairy cows fed the high concentrate (HC) and high forage (HF) group

Metabolites (μM)	Diet^1)^		p-value	VIP^2)^

	HF	HC		
Glutaric acid monomethyl ester	0.08±0.01	0.64±0.66	0.032	1.99
Lactulose	2.09±1.83	9.27±3.79	0.017	2.16
Leucine	16.85±7.72	54.32±18.22	0.001	1.92
Lactose	5.26±3.58	13.65±6.02	0.078	1.60
Acetoacetate	2.67±1.70	6.37±3.32	0.050	1.45
Glucose	286.55±41.11	318.77±35.17	0.042	0.62
Citrate	50.85±2.78	40.95±6.37	0.065	0.59
1-Methylhistidine	2.37±0.75	0.87±0.76	0.059	1.68
3-Methylhistidine	3.65±2.28	1.37±0.62	0.063	1.34
Betaine	32.47±3.84	11.65±7.15	0.012	1.70
2-Hydroxyisobutyrate	0.67±0.05	0.12±0.01	<0.001	2.29
Syringate	0.49±0.37	0.06±0.02	0.039	1.94
Carnitine	4.87±4.61	0.36±0.36	0.094	2.03

1) HF, high forage diet (10 kg; Italian ryegrass 80%: concentrate 20%); HC, high concentrate diet (14.2 kg; Italian ryegrass 20%: concentrate 80%).

2) VIP, variable importance in projection.

**Table 2:** Identification of significantly different metabolites (mean±standard deviation) in milk between dairy cows fed the high concentrate (HC) and high forage (HF) group

Metabolites (μM)	Diet^1)^		p-value	VIP^2)^

	HF	HC		
Methylsuccinate	0.84±0.13	5.10±0.77	<0.001	2.12
2-Oxoglutarate	31.27±29.25	188.05±81.52	0.018	1.87
Succinate	2.32±2.32	14.10±4.18	0.007	2.13
Glucose	82.12±0.82	350.18±180.80	0.067	1.43
1,2-Ethanediol	2,004.80±2,004.80	7,112.10±2,547.60	0.010	1.77
Urea	402.75±14.09	903.00±135.85	0.004	1.25
O-acetylcarnitine	185.50±36.64	125.40±11.99	0.009	1.14
Betaine	380.85±72.66	221.15±103.03	0.014	1.34
Arabinitol	1,761.70±508.98	827.20±683.19	0.078	1.52
Carnitine	169.65±48.30	76.69±64.76	0.093	1.54
Theophylline	2.60±0.84	1.16±0.64	0.082	1.45
Acetate	63.70±11.28	22.77±12.35	0.021	1.76
Lactulose	207.10±138.02	69.17±72.39	0.061	1.77
Alanine	28.00±9.28	9.09±9.54	0.016	1.89
N6-acetyllysine	32.57±25.63	10.43±0.34	0.093	1.33
N-acetylglutamate	21.68±17.37	6.76±0.19	0.080	1.36
Sebacate	97.80±30.68	29.13±31.78	0.007	2.01
Butyrate	66.38±70.38	14.56±0.13	0.088	1.45
Trimethylamine	3.26±1.89	0.64±0.05	0.017	1.87
Creatine	167.20±157.99	24.14±16.69	0.051	1.93

1) HF, high forage diet (10 kg; Italian ryegrass 80%: concentrate 20%); HC, high concentrate diet (14.2 kg; Italian ryegrass 20%: concentrate 80%).

2) VIP, variable importance in projection.

## SUPPLEMENTARY MATERIAL

**Supplementary Table S1.** The formulation and chemical composition of experiment diet
