# Supplementary material for: Blood and milk metabolites of Holstein dairy cattle for the development of objective indicators of a subacute ruminal acidosis
Source: Anim Biosci. 2023 Feb 26;36(8):1199–208. doi: 10.5713/ab.22.0486 (PMC10330974; doi:10.5713/ab.22.0486)
Supplement: Supplementary file 1 [file ab-22-0486-Supplementary-Table-1.pdf]

Supplementary table 1. The formulation and chemical composition of experiment diet

Content	*% of DM*	Content	Concentrate	Italian ryegrass
Ingredients		*Chemical composition (% of DM)*		
Ground corn	3.06	Moisture	11.20	7.40
Ground wheat	17.44	Crude protein	20.01	5.60
Salt	0.70	Ether extract	2.00	0.84
Molasses	3.50	Crude ash	6.43	4.84
Soy hull	5.36	Crude fiber	20.00	34.84
Wheat flour	20.10	Ca	0.80	0.21
Rice bran	0.84	P	0.51	0.13
Corn DDG	5.00	Acid detergent fiber	6.86	38.95
DDGS	15.00	Neutral detergent fiber	19.81	66.54
Soybean meal	6.12			
Urea	0.51			
Sodium bicarbonate	0.84			
Condensed molasses solubles	1.50			
Corn gluten feed	18.00			
Limestone	1.27			
Palm oil	0.39			
Flavor	0.02			
Mineral/Vitamin premix[1]	0.35			
Total	100.00			

[1]Mineral & vitamin premix contained vit. A 2,650,000 IU, vit. D3 530,000 IU, vit. E 1,050 IU, niacin 10,000 mg, Mn 4,400 mg, Zn 4,400 mg, Fe 13,200 mg, Cu 2,200 mg, iodine 440 mg, and Co, 440 mg/kg of Grobic-DC provided from Bayer Health Care (Leverkusen, Germany).

DDG: Distillers dried grains, DDGS: Distillers dried grains with solubles.
